# Supplementary material for: Mapping the evolution of fertility support policies in China: A content and instrumental analysis
Source: PLoS One. 2025 Oct 9;20(10):e0332137. doi: 10.1371/journal.pone.0332137 (PMC12510515; doi:10.1371/journal.pone.0332137)
Supplement: S1 Appendix — (ZIP) [file pone.0332137.s001.zip › S1 Appendix. 226 original policy documents/124-托育机构婴幼儿喂养与营养指南(试行).doc]

**托育机构婴幼儿喂养与营养指南**

**（试行）**

根据《国务院办公厅关于促进3岁以下婴幼儿照护服务发展的指导意见》（国办发〔2019〕15号）、《托育机构设置标准（试行）》和《托育机构管理规范（试行）》、《托儿所、幼儿园建筑设计规范（2019年版）》、《婴幼儿辅食添加营养指南》（WS/T678-2020）、《中国居民膳食指南（2016）》、《婴幼儿喂养健康教育核心信息》，我委组织编写了《托育机构婴幼儿喂养与营养指南（试行）》。

本指南适用于经有关部门登记、卫生健康行政部门备案，为3岁以下婴幼儿提供全日托、半日托、计时托、临时托等托育服务的机构。

6-24月龄婴幼儿喂养与营养要点

托育机构应与家庭配合，为实现母乳喂养提供便利条件，尽量采用亲喂母乳喂养。在母乳喂养同时为婴幼儿提供适宜的辅食。

1.支持母乳喂养。

托育机构在妇幼保健机构、基层医疗卫生机构的指导下，做好母乳喂养宣教。按照要求设立喂奶室或喂奶区域，并配备相关设施、设备。鼓励母亲进入托育机构亲喂，做好哺乳记录，保证按需喂养。

2.辅食添加原则与注意事项。

（1）从6月龄开始添加辅食，选择富含铁的泥糊状食物。

（2）鼓励尝试新的食物，每次只引入1种。留意观察是否出现呕吐、腹泻、皮疹等不良反应，适应1种食物后再添加其他新的食物。若婴幼儿出现不适或严重不良反应，及时通知家长并送医。

（3）逐渐调整辅食质地，与婴幼儿的咀嚼吞咽能力相适应，从稠粥、肉泥等泥糊状食物逐渐过渡到半固体或固体食物等。1岁以后可吃软烂食物，2岁之后可食用家庭膳食。

（4）逐渐增加食物种类，保证食物多样化，包括谷薯类、豆类和坚果类、动物性食物（鱼、禽、肉及内脏）、蛋、含维生素A丰富的蔬果、其他蔬果、奶类及奶制品等7类。

（5）辅食应选择安全、营养丰富、新鲜的食材，并符合婴幼儿喜好。婴幼儿辅食应单独制作，1岁以内婴儿辅食应当保持原味，不加盐、糖和调味品。制作过程注意卫生，进食过程注意安全。

3.自带食物管理。

如家长要求使用自带食物，托育机构应与家庭充分沟通，并做好接收和使用记录。如使用特殊医学用途婴儿配方食品，家长应提供医生或临床营养师的建议。

4.顺应喂养。

托育机构应根据不同年龄婴幼儿的营养需要、进食能力和行为发育需要，提倡顺应喂养。喂养过程中，应及时感知婴幼儿发出的饥饿和饱足反应（动作、表情、声音等），及时做出恰当的回应，鼓励但不强迫进食。从开始辅食添加起，引导婴幼儿学习在嘴里移动、咀嚼和吞咽食物，逐步尝试自主进食。

24-36月龄幼儿的喂养与营养要点

1.合理膳食。

（1）食物搭配均衡，每日膳食由谷薯类、肉类、蛋类、豆类、乳及乳制品、蔬菜水果等组成。同类食物可轮流选用，做到膳食多样化。

（2）每日三餐两点，主副食并重。加餐以奶类、水果为主，配以少量松软面点。份量适宜，不影响正餐进食量。晚间不宜安排甜食，以预防龋齿。

（3）保证幼儿按需饮水，根据季节酌情调整。提供安全饮用水，避免提供果汁饮料等。

（4）选择安全、营养丰富、新鲜的食材和清洁水制备食物。制作过程注意卫生，进食过程注意安全。

（5）食物合理烹调，适量油脂，少盐、少糖、少调味品。宜采用蒸、煮、炖、煨等方法，少用油炸、熏制、卤制等。

2.培养良好的习惯。

（1）规律进餐，每次正餐控制在30分钟内。鼓励幼儿自主进食。

（2）安排适宜的进餐时间、地点和场景，根据幼儿特点选择和烹制食物，引导幼儿对健康食物的选择，培养不挑食不偏食的良好习惯，不限制也不强迫进食。进餐时避免分散注意力。开始培养进餐礼仪。

（3）喂养过程中注意进食安全，避免伤害。不提供易导致呛噎的食物，如花生、腰果等整粒坚果和葡萄、果冻等。

（4）合理安排幼儿的身体活动和户外活动，建议户外活动每天不少于2小时。

婴幼儿食育

食育有益于身心健康，增进亲子关系。托育机构与家庭配合开展食育，让婴幼儿感受、认识和享受食物，培养良好进食行为和饮食习惯，启蒙中华饮食文化。

1.感受和认识食物。

适时引导婴幼儿感受食物，通过视觉、触觉、嗅觉、味觉、听觉等感知食物的色、香、味、质地，激发对食物的兴趣，促进认识食物，接受新食物。可以让幼儿观察或参与简单的植物播种、照料、采摘等过程，并让幼儿参与食物的制备。

2.培养饮食行为。

营造安静温馨、轻松愉悦的就餐环境，引导婴幼儿享受食物，逐步养成规律就餐、专注就餐、自主进食的良好饮食习惯。正确选择零食，避免高糖、高盐和油炸食品。

3.体验饮食文化。

培养用餐礼仪、感恩食物、珍惜食物。结合春节、元宵、端午和中秋等传统节日活动，让幼儿体验中华饮食文化。

喂养和膳食管理

1.规章制度建设。

按照《食品安全法》、《食品安全法实施条例》等要求，严格落实各项食品安全工作，强化责任意识，制定食品安全应急处置预案，做好食源性疾病防控工作。

（1）托育机构应建立完善的母乳、配方食品和商品辅食喂养管理制度和操作规范，包括喂奶室管理制度，配方食品和商品辅食的接收、查验及储存、使用制度，及相关卫生消毒制度。

（2）托育机构从供餐单位订餐的，应当建立健全机构外供餐管理制度，选择取得食品经营许可、能承担食品安全责任、社会信誉良好的供餐单位。对供餐单位提供的食品随机进行外观查验和必要检验，并在供餐合同（或者协议）中明确约定不合格食品的处理方式。

（3）鼓励母乳喂养，为哺乳母亲设立喂奶室，配备流动水洗手等设施、设备。

（4）托育机构乳儿班和托小班设有配餐区，位置独立，备餐区域有流动水洗手设施、操作台、调配设施、奶瓶架，配备奶瓶清洗、消毒工具，配备奶瓶、奶嘴专用消毒设备，配备乳类储存、加热设备。

（5）托育机构应配备食品安全管理人员，并制订食堂管理人员、从业人员岗位工作职责，食品安全管理人员及从业人员上岗前应当参加食品安全法律法规和婴幼儿营养等专业知识培训。

（6）婴幼儿膳食应有专人负责，班级配餐由专人配制分发，工作人员与婴幼儿膳食要严格分开。

（7）做好乳类喂养、辅食添加、就餐等工作记录。

2.膳食和营养要求。

食品应储存在阴凉、干燥的专用储存空间。标注配方食品的开封时间，每次使用后及时密闭，并在规定时间内食用。配方食品应按照产品使用说明按需、适量调配，调配好的配方奶1次使用，如有剩余，直接丢弃。配方食品在规定的配餐区完成。调配好的配方奶，喂养前需要试温，做好喂养记录。

（1）托育机构应根据不同月龄(年龄)婴幼儿的生理特点和营养需求，制定符合要求的食谱，并严格按照食谱供餐。

（2）食谱按照不同月龄段进行制定和实施，每1周或每2周循环1次。食谱要具体到每餐次食物品种、用量、烹制或加工方法及进食时间。

（3）主副食的选料、洗涤、切配、烹调方法要适合不同月龄（年龄）婴幼儿，减少营养素的损失，符合婴幼儿清淡口味，达到营养膳食的要求。烹调食物注意色、香、味、形，提高婴幼儿的进食兴趣。

（4）食谱中各种食物提供的能量和营养素水平，参照中国营养学会颁布的《中国居民膳食营养素参考摄入量(DRIs)(2013)》推荐的相应月龄(年龄)婴幼儿每日能量平均需要量(EER)和推荐摄入量(RNI)或适宜摄入量(AI)确定。

（5）食谱各餐次热量分配：早餐提供的能量约占一日的30%(包括上午10点的点心)，午餐提供的能量约占一日的40%(含下午3点的午后点)，晚餐提供的能量约占一日的30%(含晚上8点的少量水果、牛奶等)。

（6）食谱中各种食物的选择原则以及食物用量，参照中国营养学会颁布的《7—24月龄婴幼儿喂养指南(2016)》、《学龄前儿童膳食指南(2016)》中膳食原则，以及《7-24月龄婴幼儿平衡膳食宝塔》、《学龄前儿童平衡膳食宝塔》中建议的食物推荐量范围。

（7）半日托及全日托的托育机构至少每季度进行一次膳食调查和营养评估。提供一餐的托育机构(含上、下午点)每日能量和蛋白质供给量应达到相应建议量的50%以上；提供两餐的托育机构，每日能量和蛋白质供给量应达到相应建议量的70%以上；提供三餐的托育机构，每日能量和蛋白质和其他营养素的供给量应达到相应建议量的80%以上。

（8）三大营养素热量占总热量的百分比是蛋白质12～15%，脂肪30～35%，碳水化合物50～65%。优质蛋白质占蛋白质总量的50%以上。

（9）有条件的托育机构可为贫血、营养不良、食物过敏等婴幼儿提供特殊膳食，有特殊喂养需求的，婴幼儿监护人应当提供书面说明。

（10）定期进行生长发育监测，保障婴幼儿健康生长。
